# Examining Demographic Characteristics of Firearm Owners Currently Engaged in Mental Health Treatment

**DOI:** 10.1002/jclp.70048

**Published:** 2025-10-05

**Authors:** Allison E. Bond, Taylor R. Rodriguez, Kimberly Burke, Sultan Altikriti, Michael D. Anestis

**Affiliations:** ^1^ The New Jersey Gun Violence Research Center Piscataway New Jersey USA; ^2^ Department of Urban‐Global Public Health Rutgers University Piscataway New Jersey USA

**Keywords:** adult mental health, firearm owners, mental health treatment, psychiatric medications, suicide, suicide prevention

## Abstract

**Objective:**

Research on the demographic characteristics and mental health profiles of those with firearm access is scarce. To address this gap, the current study examined the demographic characteristics and use of mental health services among firearm owners in the United States.

**Methods:**

Using a sample of 3018 US adults with firearm access drawn from a nationally representative sample of adults (*n* = 8009), this study assessed the relationships between individual characteristics, engagement in therapy, and receiving prescribed psychiatric medication among respondents who have access to firearms.

**Results:**

Among those with firearm access, being younger, female, having higher education, and a history of suicidal ideation were associated with engagement in therapy and receiving psychiatric medication. Additionally, being nonwhite and employed were associated with lower odds of receiving medication. The findings highlight the need to better identify high‐risk subgroups (e.g., nonwhite, older, and less educated) with firearm access who do not engage with mental health services.

**Conclusion:**

It is hypothesized that extending mental health services and suicide prevention strategies to those who traditionally underutilize these services despite an elevated risk of self‐harm can help reduce self‐injury and potentially reduce firearm‐related suicides among these populations.

## 1 Introduction

The presence of a firearm in the home increases the risk of injuries and death, such as suicide, for every member of that home (Anglemyer et al. 2014). In fact, firearms are the most common method for suicide (Center for Disease Control and Prevention [CDC] 2025). There have been efforts to reach firearm owners with safety resources, and to provide interventions such as lethal means counseling to mitigate such risks (Office of the Surgeon General 2024). One avenue to reach firearm owners, especially those at risk for suicide, is through mental healthcare systems as those at risk for suicide may present for psychological treatment. The United States' surgeon general's recent report discusses the importance of connecting firearm owners and those impacted by firearm violence to mental healthcare (Office of the Surgeon General 2024), this is not to suggest that all firearm owners require mental health care services, but that it can be a useful tool to any individual—regardless of firearm ownership status—exposed to violence or at risk for suicide. Despite this emphasis, research has demonstrated that many individuals who die by suicide with a firearm do not utilize mental healthcare services (Bond et al. 2022). However, there is limited literature identifying subgroups of firearm owners that may be more or less likely to utilize services. (Table 1).

**Table 1:** Sample characteristics.

	Total sample	Individual therapy	Psychiatric medication
	*N* = 3018	*N* = 197	*N* = 167
Age			
Mean (SD)	49.85 (17.717)	39.93 (14.049)	40.75 (15.214)
Range	18–98	18–84	18–81
Race			
White	2189 (72.5%)	141 (71.7%)	131 (78.3%)
Other	823 (27.5%)	56 (28.3%)	36 (21.7%)
Sex			
Male	1547 (51.3%)	78 (39.3%)	58 (34.5%)
Female	1471 (48.7%)	120 (60.7%)	110 (65.5%)
Education			
No High School Degree	226 (7.5%)	18 (9.0%)	13 (7.8%)
High School Degree	902 (29.9%)	41 (20.8%)	37 (21.9%)
Associate's Degree	910 (30.1%)	60 (30.6%)	57 (34.2%)
Bachelor's Degree	582 (19.3%)	42 (21.5%)	35 (21.0%)
Master's Degree or higher	398 (13.2%)	36 (18.1%)	25 (15.1%)
Household Income			
< $10,000	49 (1.6%)	4 (2.2%)	4 (2.2%)
$10,000–$24,999	138 (4.6%)	17 (8.6%)	8 (4.6%)
$25,000–$49,999	380 (12.6%)	23 (11.7%)	29 (17.3%)
$50,000–$74,999	493 (16.3%)	27 (13.5%)	22 (13.3%)
$75,000–$99,999	434 (14.4%)	27 (13.6%)	19 (11.4%)
$100,000–$149,999	685 (22.7%)	45 (22.9%)	47 (27.8%)
$150,000+	840 (27.8%)	54 (27.5%)	39 (23.4%)
Marital Status			
Currently Not Married	1179 (39.1%)	109 (55.3%)	90 (53.6%)
Currently Married	1838 (60.9%)	88 (44.7%)	78 (46.4%)
Employment Status			
Not Employed	1098 (36.4%)	63 (32.0%)	71 (42.2%)
Employed	1920 (63.6%)	134 (68.0%)	97 (57.8%)
Individual + Medication	258 (8.5%)	—	—

Within the treatment utilization literature for the general population, it is evident that several demographic factors are associated with mental health treatment seeking rates. For instance, those of minoritized identities (e.g., Black and Hispanic) report less experience with mental healthcare compared to white individuals (e.g., Alegría et al. 2002; Rodriguez et al. 2024; Yang et al. 2020). Factors such as structural barriers, institutionalized racism, stigma, and a lack of access to culturally informed care makes it more difficult for minoritized individuals to utilize treatment. Understanding treatment utilization rates for racially minoritized firearm owners is particularly important given the increasingly elevated rates of firearm‐suicide and homicide deaths within these communities (Centers for Disease Control and Prevention CDC 2022).

In addition to race, other sociodemographic variables such as education and socioeconomic status (SES) or household income are associated with mental healthcare service utilization. Specifically, lower levels of educational attainment and SES are associated with less service utilization (Packness et al. 2017; Zwaanswijk et al. 2003), which can be attributed to the structural barriers that can come from the inability to afford care, lack of insurance, or less mental health literacy. It is likely that income‐based differences may also extrapolate to employment status. Those who are unemployed may have lower household income and, thus, may be less likely to have access to mental healthcare. Furthermore, those with access to health insurance are more likely to utilize mental healthcare (Walker et al. 2015). It is plausible that without access to employer provided insurance, those who are unemployed face more structural barriers to treatment.

Sex, age, and marital status also impact treatment utilization. Females are typically more likely to engage with mental healthcare, which can, in part, be attributed to the tendency for females to have more positive attitudes toward treatment (Oliver et al. 2005; Wendt and Shafer 2016). Less positive treatment attitudes among men can result in more attitudinal barriers to utilization and ultimately less engagement in services. Regarding age, mental healthcare utilization seems to depend on the type of services investigated. Older adults are less likely to engage in mental healthcare with a specialist (e.g., psychologist) than younger adults, instead preferring to garner mental healthcare from medical providers such as a primary care doctor (Crabb and Hunsley 2006; Mackenzie et al. 2006). These findings suggest that, especially for age‐based findings, mental healthcare utilization rates may differ when examining psychotherapy versus psychiatric medications. Compared to other sociodemographic variables, there has been less attention to marital status in the context of mental healthcare utilization. There are mixed findings regarding the associations between marital status and mental health overall, which makes it difficult to ascertain whether marital status impacts treatment utilization (Williams et al. 2010). Some findings suggest that married individuals have improved mental health, as such their need for services may dissipate. On the other hand, the presence of marital discord may increase mental health difficulties and treatment utilization. Similarly, individuals react differently to divorce, which may differentially impact treatment seeking and utilization rates.

In addition to demographic factors, existing mental health concerns may impact treatment utilization. Specifically, determining whether those with suicidal thoughts are more or less likely to seek treatment is vital for reducing the firearm suicide rate. Within military populations, help‐seeking rates are higher for those with suicidal thoughts compared to those with other psychological difficulties (Hom et al. 2017). These findings are promising, as they suggest that those thinking of suicide are connected to services, however, these findings are not specific to firearm owners and have been limited to military specific samples. As such, research has yet to examine how such findings may extrapolate to firearm owners in general.

The present study seeks to identify the characteristics of firearm owners who are currently involved in mental healthcare services. We examine demographic group differences between those who are and are not in individual psychotherapy and psychiatric medication. Demographics included are racial identity, sex, age, education level, marital status, employment status, household income, and self‐reported history of suicidal ideation. In line with extant research, we expect for individuals who are in treatment to more likely be white, female, younger, of higher education and higher household income, employed, and to report suicidal thoughts in their lifetime. We do not have a priori hypotheses for marital status, given the lack of extant literature. Similarly, we are unaware of other research to examine treatment utilization rates by type of services (i.e., individual therapy vs. psychiatric medication) in this population. However, given older adults’ preferences for medical doctors to provide mental healthcare, we anticipate that older adults will be less likely to utilize psychotherapy but more likely to utilize psychiatric medication compared to younger adults. Findings from the present study will add to our understanding of the subgroups of firearm owners that mental healthcare providers may encounter and could benefit from firearm safety efforts and which firearm owners may need to be reached through other channels.

## 2 Methods

### 2.1 Participants

The data constitute a subset of firearm owners (*n* = 3,018) from a nationally representative sample of adults living in the US recruited via Ipsos KnowledgePanel (KP), a nationally representative polling service, between May 15 and May 28, 2024. To arrive at this sample, individuals from the KP respondent pool were randomly emailed an invitation to complete the survey. Of the *n* = 8647 respondents, *n* = 8009 completed the survey (93%), and *n* = 3018 of whom had access to a firearm. To ensure that the sample is population‐representative, sample weights were created using geodemographic distributions from the March 2023 supplement of the Current Population Survey and the Census Bureau's American Community Survey. Specifically, the KP procedure used an iterative proportional fitting process to create sampling weights to match known population distributions of sex, race/ethnicity, census region, education level, household income, and political identification. A previous analysis found Ipsos KnowledgePanel to be superior to another mainstream alternative (Herman et al. 2024). Weights were used in all analyses to ensure national representativeness. Data may be made available to other qualified researchers (e.g., advanced degree in relevant field and a history of scholarship. If interested, please contact the corresponding author with the request.

### 2.2 Measures

#### 2.2.1 Firearm Access and Demographics

Firearm access and demographic variables were developed by the research team and have been used in multiple other published studies. Firearm access was measured by asking respondents, “Is there typically a firearm or firearms stored in or around your home?” This measure does not exclusively capture firearm ownership and is helpful in capturing both owners and those who have access to a firearm in their home. This is important given that firearm access, not only ownership, increases the risk of suicide. Demographic variables were derived from KP profiles. Race and sex were assessed with single‐item categorical measures. Income^1^, education level, and employment status were assessed with single item continuous measures.

#### 2.2.2 Individual Therapy and Medication

Engagement in individual therapy was coded as 0 = *no* and 1 = *yes* if the respondent endorsed receiving individual therapy to the question are “you currently receiving mental health services from a professional, like a psychologist or a psychiatrist?” The use of psychiatric medication was assessed based on the same question with respondents endorsing 0 = *no* or 1 = *yes* for receiving psychiatric medication.

#### 2.2.3 Suicidal Ideation

Consistent with prior research, lifetime suicidal ideation was assessed using eight items from the Self‐Injurious Thoughts and Behaviors Interview–Short Form–Self Report (SITBI‐SF‐SR; Fox et al. 2020). Participants were asked, “Have you ever had any of the following thoughts for more than a few minutes?” followed by eight examples of suicidal ideation (e.g., ‘I wish I were dead,’ ‘I should kill myself,’ and ‘I wish I could go to sleep and never wake up’). Participants then endorsed any of the eight that they had experienced. These responses were dichotomized to indicate the presence or absence of any lifetime suicidal ideation (0 = *no suicidal ideation*, 1 = *any suicidal ideation*). We use lifetime suicidal ideation to ensure that all of the suicidal ideation treatment‐relevant history is captured in this measure.

#### 2.2.4 Data Analytic Plan

Descriptive statistics were generated for all variables of interest. Two logistic regression analyses were conducted to examine demographic differences among (1) firearm owners currently receiving individual therapy and (2) firearm owners receiving psychiatric medication. For both models, the following variables were used as predictors: race (White vs. nonwhite), sex, age, education level, marital status, employment status, household income, and lifetime suicidal ideation.

## 3 Results

### 3.1 Firearm Owners

As can be seen in Table 2, firearm owners who were currently in individual therapy were more likely to be female (*p* = 0.009, OR = 1.509 [1.110, 2.051]), younger (*p* < 0.001, OR = 0.971 [0.961, 0.981]), have higher education (*p* < 0.001, OR = 1.368 [1.180, 1.584]), and report lifetime suicidal ideation (*p* < 0.001, OR = −3.071 [2.241, 4.028]) compared to those not in individual therapy.

**Table 2:** Logistic regression examining demographic differences in individual therapy among firearm owners.

	P	OR	CI	
White	0.762	1.053	0.753	1.473
Sex	0.009	1.509	1.110	2.051
Age	< 0.001	0.971	0.961	0.981
Education	< 0.001	1.368	1.180	1.584
Marital	0.052	0.715	0.510	1.003
Employment	0.188	0.793	0.561	1.120
Household income	0.402	0.957	0.864	1.060
Suicidal ideation	< 0.001	3.071	2.241	4.208

As can be seen in Table 3, firearm owners who were currently receiving psychiatric medication were more likely to be white (*p* = 0.040, OR = 1.511 [1.019, 2.242]), younger (*p* < 0.001, OR = 0.972 [0.962, 0.983]), female (*p* = 0.001, OR = 1.758 [1.250, 2.470]), have higher education (*p* < 0.001, OR = 0.489 [0.341, 0.701]), and report lifetime suicidal ideation (*p* < 0.001, OR = 4.078 [2.902, 5.730]) compared to those not receiving medication. Additionally, those who received medication were less likely to be employed (*p* < 0.001, OR = 0.489 [0.341, 0.701]).^2^, ^3^

**Table 3:** Logistic regression examining demographic differences among firearm owners receiving psychiatric medication.

	P	OR	CI	
White	0.040	1.511	1.019	2.242
Sex	0.001	1.758	1.250	2.470
Age	<0.001	0.972	0.962	0.983
Education	<0.001	1.347	1.147	1.582
Marital	0.165	0.771	0.534	1.113
Employment	<0.001	0.489	0.341	0.701
Household income	0.695	0.978	0.875	1.093
Suicidal ideation	<0.001	4.078	2.902	5.730

## 4 Discussion

The present study aimed to identify demographic characteristics of firearm owners currently engaged in psychotherapy or psychiatric medication services. Consistent with our hypotheses, the findings indicated that younger age, females, higher education, and a history of lifetime suicidal ideation were associated with receiving psychotherapy. Additionally, being white, females, having higher education, and reporting lifetime suicidal ideation were associated with increased odds of receiving psychiatric medication. Contrary to our expectations, older age was not significantly associated with receiving psychiatric medication. Furthermore, no significant differences emerged in terms of marital status or income in either analysis. These results highlight that demographic factors, such as sex, race, education level, employment status, and history of suicidal ideation play a role in determining if firearm owners engage in mental health services.

Findings indicate that female firearm owners are more likely than male firearm owners to receive both individual therapy and psychiatric medication. Several factors may contribute to this disparity, including the stigma surrounding mental healthcare. Specifically, research suggests that men are less likely to acknowledge the effectiveness of individual therapy (Pattyn et al. 2015), which may lead to lower rates of seeking therapy due to a perception that it is unhelpful. A similar trend is observed with psychiatric medication, where female firearm owners are more likely to receive medication compared to their male counterparts, a pattern that aligns with the general population (Johansen 2021). One possible explanation for these findings is that males, in general, seek treatment at lower rates than females (e.g., Liddon et al. 2018) and therefore are less likely to be referred for individual therapy and to be prescribed psychiatric medications. Additionally, adherence to traditional masculine norms and sex‐based stereotypes may discourage men from pursuing treatment, and this may be particularly salient among those who own firearms. Firearm owning men often exhibit strong identification with masculinity, which influences both their reluctance to seek mental health services and their firearm ownership and storage behaviors (Daruwala et al. 2023). Concerningly, though, men are more likely to own firearms than women (Miller et al. 2022) and are more likely to use a firearm in a suicide death. Findings from this study highlight that men who own firearms are less likely to seek treatment than women who own firearms. Therefore, promoting treatment seeking among men is an important suicide prevention and gun violence prevention strategy. Firearm suicide prevention strategies, such as secure firearm storage and secure storage maps, may be more effective for male firearm owners when they are disseminated in non‐health care settings where male firearm owners frequently interact, such as barbershops and shooting ranges.

White firearm owners were more likely to receive psychiatric medication than racial and ethnic minorities. This finding aligns with broader systemic barriers to care and implicit biases in healthcare that influence treatment outcomes. Specifically, it is well documented that racial and ethnic minorities receive poorer quality healthcare compared to white individuals (Odonkor et al. 2021). This disparity may lead to racial and ethnic minorities being prescribed psychiatric medication at lower rates than their white counterparts. Research has also shown that, when Black individuals seek mental healthcare, they report more unfavorable encounters than white individuals (Diala et al. 2000), and Black females often terminate treatment early due to a lack of cultural competency from providers (Mengesha and Ward 2012). Given that this study assessed current psychiatric medication, it is possible that some racial and ethnic minorities previously sought psychiatric medication but discontinued due to negative experiences in healthcare. Given the systemic barriers to care that racial and ethnic minority individuals face, combined with the stigma surrounding firearm ownership, racial and ethnic minority firearm owners may experience even more barriers to seeking mental health care. This is concerning given the rising firearm suicide rates among Black men and other minority subgroups (CDC 2025). For these communities, cultural stigma around both mental health care and firearm ownership may make it even harder to access the support they need. Findings from this study highlight the need to increase culturally competent mental healthcare that addresses the unique experiences of minority firearm owners. In addition, there is a need to reduce implicit biases within the healthcare system and eliminate disparities in treatment access for racial and ethnic minority firearm owners. This may improve mental health outcomes overall and reducing the rising firearm suicide rates.

The findings around other demographic factors, such as marital status, age, and socioeconomic variables, show that their impact on service utilization is more nuanced than initially expected. Among firearm owners, marital status did not significantly impact receiving individual therapy or receiving psychiatric medication. One potential explanation for this finding is that marital dynamics vary among couples, which makes it difficult to generalize the impact of being married versus not being married on service utilization. In terms of age, those who were younger were more likely to receive individual therapy and psychiatric medication than those who are older. This is consistent with previous research indicating that stigma surrounding mental health care is higher among older adults than younger adults (Klap et al. 2003), which may contribute to the younger individuals seeking treatment at higher rates. Additionally, older adults often face more physical health concerns, which may limit their ability to be prescribed psychiatric medications due to interactions with other medications or the medications negatively impacting their physical health. It may also be that currently, younger generations are experiencing heightened rates of mental health related concerns which drive help‐seeking behaviors. Measures of socioeconomic status, including income, employment, and education, showed sporadic findings. Specifically, income level was not significantly associated with treatment utilization. However, those with higher education were more likely to receive individual therapy, and those with higher education who were employed were more likely to receive psychiatric medication. Education is often associated with income status, with those who have higher education and those who are employed generally having higher incomes than those with lower education. Therefore, income may not have been significant because it was already accounted for by education. The findings around education are in line with previous research, highlighting that those with higher education are more likely to seek treatment (Steele et al. 2007). The findings related to marital status, age, and socioeconomic factors among firearm owners seeking mental health treatment largely mirror those of the general population. While this similarity is expected, it's important to consider what these patterns mean for firearm owners. Older firearm owners are less likely to seek treatment compared to younger owners, despite being more likely to use a firearm in a suicide death. Additionally, those with higher education levels were more likely to seek mental health care. However, states with lower educational attainment—such as Mississippi (U.S. Census Bureau 2025)—also have some of the highest firearm injury and fatality rates (CDC 2025), indicating that those who are at risk for firearm suicide may not have access to care. Reducing barriers to mental health treatment for older adults and individuals with lower education levels could positively impact firearm suicide rates and improve the quality of life for those at risk.

Lifetime suicidal ideation was significantly associated with receiving individual therapy and psychiatric medications. While the finding indicating that firearm owners who are experiencing thoughts of suicide are connected with mental health care is promising, it is important to consider this within the context of suicide risk among firearm owners broadly. Overall, the vast majority of those who experience thoughts of suicide do not go on to attempt or die by suicide. However, only 26.6% of those who died by suicide using a firearm have ever sought mental health or substance use treatment (Bond et al. 2022), indicating that the vast majority of those who die by firearm suicide do not interact with mental health care. Therefore, firearm owners who are experiencing suicidal ideation and seeking mental health care may not be representative of those who will ultimately die by firearm suicide. While it is important to ensure those with suicidal ideation are provided evidence‐based treatments to help reduce their thoughts of suicide and improve their lives, it is equally important to identify firearm owners who are at risk for dying by suicide and do not interact with mental health care. It is important to note that the presence of a firearm in the home increases the risk of suicide and makes it more likely that an individual experiencing suicidal ideation will act on those thoughts in a lethal way (Nguyen et al. 2024). Additionally, the environment and information consumed by firearm owners (e.g., their infosphere) may influence how they view mental health treatment and what steps they are willing to take to reduce suicide risk (Vuong et al. 2025). This is closely related to secure firearm storage. For example, if a firearm owner consistently receives messages suggesting that responsible ownership means keeping a firearm readily accessible for protection, they may be less likely to engage in secure storage for suicide prevention. Understanding where individuals seek help before dying by suicide is therefore critical for developing targeted interventions, such as lethal means counseling. It also highly important to ensure that accurate, health‐promoting information reaches the right audiences through trusted and effective channels.

Overall, the demographic characteristics associated with mental healthcare utilization in this study are consistent with previous research on the general population. However, firearm owners and firearm suicide decedents do not demographically align with the general population, as white men die by suicide at higher rates than other groups. These findings suggest that while some demographic subgroups of firearm owners seek mental health treatment, those at highest risk for firearm suicide are less likely to engage in services. As a result, suicide prevention and lethal means safety interventions may need to extend beyond mental healthcare. Specifically, educating trusted community members—such as faith leaders—on how to assess for suicide risk and engage discussions on lethal means counseling can help ensure that those who are not presenting to mental health care are still able to receive information on secure storage. Additionally, as can be seen in this study, healthcare providers are interacting with firearm owners who may not fit the demographic profile typically associated with firearm ownership. It is crucial to screen all clients for firearm access and discuss secure firearm storage with firearm owners, regardless of demographics.

While informative, the present study is not without its limitations. First, we assessed for current individual therapy and psychiatric medication, and therefore are not able to capture lifetime engagement in services. In line with this, we were also not able to capture the specific types of medication of psychotherapy firearm owners engaged in, and did not assess for psychiatric diagnoses which may have motivated individuals for treatment. It is possible that demographic subgroups who seek treatment over their lifetime may differ than those who are seeking it at a single timepoint. Future research should seek to better understand lifetime treatment seeking among firearm owners. Additionally, this study was not able to capture how factors that may influence treatment seeking such as depressive symptoms and substance use differ among firearm owners and non‐firearm owners. Race was collapsed into white and other. While this was necessary due to the limited sample sizes of racial and ethnic minorities, it does not allow us to understand how treatment seeking differs among different racial and ethnic subgroups. Research that includes multiple racial categories would be a strong contribution to the existing literature. Lastly, we assessed access to a firearm, and not specifically firearm ownership. It is important to note that access to a firearm is associated with increased risk for suicide. While this approach allowed us to capture a broader range of individuals at risk for firearm injury and death, it is not without its limitations. It may be that those who have access to a firearm are demographically different than those who directly own the firearm. Research that examines demographic differences in who owns a firearm and who has access to a firearm would be a valuable contribution to the literature.

The present study identified demographic characteristics of firearm owners seeking psychotherapy or psychiatric medication services. Females, white individuals, those who have higher education and are employed, and those with lifetime suicidal ideation are more likely to seek mental healthcare. However, there are discrepancies between those who seek mental healthcare and those who are at high risk for firearm suicide. This highlights the need for suicide prevention and lethal means counseling interventions to occur outside of the mental healthcare setting.

## Data Availability

The data that support the findings of this study are available from the corresponding author upon reasonable request.

## Endnotes

1 Of note, income was treated as a continuous variable.

2 Non‐firearm owners who were currently in individual therapy were more likely to be female (*p* < 0.001, OR = 1.486 [1.211, 1.823]), younger (*p* < 0.001, OR = 0.977 [0.971, 0.984]), have higher education (*p* < 0.001, OR = 1.212 [1.099, 1.337]), be employed (*p* = 0.001, OR = 0.689 [0.550, 0.863]), and report lifetime suicidal ideation (*p* < 0.001, OR = 3.821 [3.115, 4.687]). Non‐firearm owners who were currently receiving psychiatric medication were more likely to be white (*p* < 0.001, OR = 1.514 [1.208, 1.898]), female (*p* = 0.022, OR = 1.298 [1.038, 1.623]), younger (*p* < 0.001, OR = 0.984 [0.977, 0.991]), to be married (*p* = 0.037, OR = 0.750 [0.687, 0.984]), and report lifetime suicidal ideation (*p* < 0.001, OR = 4.434 [3.535, 5.563]) compared to those not receiving medication.

3 To examine if demographic factors predict engagement in treatment to a greater degree than firearm ownership status, several post‐hoc analyses were conducted. Female non‐firearm owners were significantly more likely to seek individual treatment (12.1% vs. 8.2%, *x* = 18.675, *p* < 0.001) and receive medication (9.3% vs. 7.5%, *x* = 7.655, *p* = 0.022) than female firearm owners. Those with a history of suicidal ideation and who did not own a firearm were significantly more likely to seek individual therapy (23.0% vs. 16.6%, *x* = 9.441, *p* = 0.009) but did not significantly differ in medication (19.6% vs. 15.8%, *x* = 4.376, *p* = 0.112) than firearm owners with ideation. White non‐firearm owners were significantly more likely to receive medication (9.0% vs. 6.0%, *x* = 19.238, *p* < 0.001) than white firearm owners. Those who own and do not own firearms and are not employed did not significantly differ in terms of medication (8.3% vs. 6.5%, *x* = 4.737, *p* = −0.94). There was no significant difference between age of firearm owners and non‐firearm owners when seeking therapy (*F* = 0.321, *p* = 0.725) or medication (*F* = 0.142, *p* = 0.868). There was no significant difference in terms of education among firearm owners and non‐firearm owners when seeking therapy (*F* = 0.700, *p* = 0.497) or medication (*F* = 1.235, *p* = 0.292).
